# Annotations

**Published:** 1901-02-09

**Authors:** 


### ANNOTATIONS.

### German Measles.

It would seem not inappropriate at the present moment to recall some of the symptoms and some of the peculiarities of the disease which has by a strange freak in nomenclature has got for itself the name of German Measles. Surely no disease ever had a greater multitude of synonyms! Rötheln, German Measles, Epidemic Roseola, Bastard Measles, Epidemic Rose Rash, Rubeola Notha, which is the official name given to it in the Nomenclature of Diseases, and finally Rubella, which is the name now given to it by almost all medical men. Among all these various designations practically the choice has fallen upon Rubella as its medical, and German Measles as its popular, name.

In some particulars we have in this disease a sort of mimicry of measles, and in others a very fair imitation of scarlet fever, so much so that at one time it was commonly regarded as a hybrid disease. This, however, is a hypothesis which has long been given up, although probably there are still some who hold to it. There can be no doubt that rubella is a definite and distinct disease, differing from both measles and scarlet fever, and showing its individuality by the facts that, while one attack protects a child from a repetition of the disease, an attack of rubella does not give protection from either of the former maladies, and neither do they protect a child from being attacked in turn by rubella. The disease is highly infectious from person to person, but there seems to be no proof that the infection can be retained by the clothes. The period of incubation is not so exactly marked as is some other of the infectious fevers. Probably seventeen or eighteen days may be taken as the usual interval between exposure to infection and the appearance of the rash, but in some cases the time is a good deal shorter.

As in the case of measles a patient is infectious to others two or three days before the rash comes out, and during this time he may seem quite well, so that there is even more difficulty than with measles in checking an epidemic by isolating those attacked. There is some difference among authorities as to the length of time which may elapse between the commencement of the illness and the appearance of the rash, but there is no doubt that in the great majority of cases it does not exceed twelve hours; thus the sudden onset of rubella is markedly different from the gradual development of true measles (morbilli), which may last for three or four days before the rash appears. As to the rash, in typical cases it is characteristic enough; but all cases are not typical, and it must happen in the practice of everyone that cases will occur in which diagnosis from the rash alone is impossible. Especially does rubella tend to look like scarlet fever when, along with fever and some sore throat, the rosy-red dots run together into a sheet of roseola-like eruption. There is generally an absence of those catarrhal symptoms which are so common in true measles, although there may be cough and some signs of bronchitis, which rapidly disappear when the rash comes out. On the other hand, there is often some soreness of the throat with enlargement of the tonsils and general redness of the palate and pharynx, which may suggest scarlet fever. An important point in diagnosis is the enlargement of the glands, especially the posterior cervical glands. The enlargement of these glands is almost always an early symptom, and may be detected sometimes several days before the appearance of the rash.

Take it altogether, the disease is a mild one: although it is sometimes very uncomfortable from the burning and itching of the rash and from the fever, which is occasionally pretty high, and in uncomplicated cases its mortality is nil. It may be of interest to mention that the name of the disease does not even appear in the new tables of mortality just drawn up by a committee of the Society of Medical Officers of Health. It must be remembered, however, that it is a fever, and that a period of fever is always a period of risk. In some cases the disease has been followed by pneumonia, and perhaps more often the illness has seemed to give the opportunity for the onset of tuberculosis, or at any rate for a latent tuberculosis to spring into activity. People are apt to think very lightly of slight fevers, and of what they are pleased to call the trivial diseases of childhood. But the more carefully one searches into the past history of cases of serious disease—such as disorders of nutrition, spinal curvatures, anæmias, and the various forms of tuberculosis —the more often do we find that their starting-point has been a neglected attack of some "trivial" fever. Hence, like all fevers, even rubella should be dealt with carefully and treated with due respect.

After an isolated attack of rubella occurring in a place where there are many susceptible people round about, such as a first case in a school after the holidays, it may be well to maintain the rule that the patient should not mix with others until three weeks have elapsed from the commencement of the illness. It may be mentioned, however, that in the Code of Rules issued by the Medical Officers of Schools Association the time is given as "not less than ten days from the date of the appearance of the rash."

### Fresh Air and Influenza.

The recent signs of a recrudescence of influenza render it desirable to draw attention to the necessity for ventilation of the sick-room when dealing with that disease. Again and again it has been noticed how largely the disease tends to spread through houses and among people whose occupations keep them indoors, and how comparatively lightly the disease has fallen upon outdoor workers. Indeed, in the face of some of our recently-gained knowledge in regard to the accessory circumstances which in some cases appear necessary for the full propagation of certain forms of infection, it seems by no means certain that influenza will not have to be reckoned among the "house diseases." At any rate the tendency to spread through ill-ventilated houses is very marked, as also is its liability to take on a peculiarly virulent form where the sufferers are not furnished with sufficient air in their sick-rooms, and this is a matter of special importance in winter influenza in consequence of the dread so many people have of open windows. It is probable that in all cases of influenza the respiratory apparatus is attacked, and it is certain that in very many it is on the organs of respiration that the main stress of the disease falls. Hence among those who retain the old-fashioned dread of fresh air, and whose first instinct on feeling a "sniffle" is to shut every window as tightly as possible, the very thought of breathing fresh air while lying in bed with influenza is repugnant in the highest degree. Nevertheless there can be but little doubt that of all means for allaying many of the discomforts and avoiding many of the dangers of influenza a comfortable bed, an open window, and a good fire are among the most effectual. Unhappy Londoners, who cannot let in fresh air without admitting also an avalanche of discordant "music," a stream of smuts, and an unending procession of irritating street cries, are much to be pitied. Perhaps when it comes to be recognised, as surely it will do some time, that the sick as well as the healthy have a right to the enjoyment of the air, something will be done to lessen the abominable noises without suffering from which it is impossible for the ordinary Londoner to open his windows or to breathe fresh air in time of sickness.

### The Suppression of Quackery.

THE Americans are a practical people who like to go to the root of things. Now quackery is a thing which we, just as much as they, would like to see rooted up; but this eradicating process is the duty of no one in particular, hence no one undertakes the work. There is a strong feeling among medical men, especially among those who, having attained success and planted themselves in pleasant places, are called our professional "leaders," that to touch pitch is to be defiled, and that quackery is best left alone. Needless to say there are plenty of others who do not agree with them. But then they very often are not "leaders," and an uncharitable public is always willing to believe, and not entirely unwilling to assert, that it is envy that is at the bottom of their public spirit. Hence they also are rendered somewhat shy of tackling this blatant evil. The Medical Society of the County of New York, however, takes a more practical view of the matter. They recognise that the meaning and intention of State registration is to afford some security to the people that all who practise medicine shall at least possess some knowledge of their work, and they see that this security is entirely undermined when people—ignorant and unqualified quacks—are allowed to practise with impunity. The Society has therefore resolved to take the matter into its own hands and to begin an active crusade against these unlicensed, illegal, and incompetent practitioners. We believe that the law on the subject is much more stringent, so far as its letter goes, in New York than it is with us. But whether here or there, the efficacy of many laws depends much on the energy of those by whom they are put in force, and upon the extent which they are supported by public opinion. And here, at any rate, public opinion is all in favour of letting every man die in his own way. Sometimes, it is true, when parents stretch their parental rights to the extent of deciding by what particular form of quackery, or laying-on of hands, or what not, their children shall be removed to happier spheres, a British jury can be found to summon up courage to raise a mild protest. But take it all in all, we are a tolerant people. Public opinion is in favour of free trade in quackery, and if satisfied that a patient is kindly treated and decently fed cares little whether our redundant population is diminished by aid of Christian science, quack medicines, or paregoric. If our medical societies were to try to intervene, we fear they would only find themselves crying in the wilderness.

### Was it Arsenic after all?

DR. TUNNICLIFFE,[1] of King's College, and Dr. Otto Rosenheim, Ph.D., insist again upon what has been pointed out before, namely, the incongruity between the amount of arsenic taken by many of the sufferers in the recent epidemic of beer-poisoning in the Manchester district and the intensity of the effects produced. The explanation offered by the authors is, that the symptoms may not have been due to arsenic alone, but that some tangible poisonous substance which has up to the present escaped detection and estimation has found its way into the beer along with the arsenic. In searching for such a substance, they directed their attention to the possible impurities of sulphuric acid, other than arsenic, but exerting a physiological action similar to it, and selenium and thallium at once suggested themselves. It is a known fact that selenium occurs together with arsenic in nearly all pyrites and in the sulphuric acids prepared from them. They were able to convince themselves that even colourless commercially-purified English sulphuric acid still contained selenium, and that the dark brown commercial "double rectified oil of vitrol" (D.O.V. or R.O.V.) as sent out by the chemical manufacturer implicated in the recent poisoning cases, and therefore probably identical with that alleged to have been ordered by the sugar manufacturers, gave a very strong selenium (and arsenic) reaction. The chemical properties of selenium and selenious acid render it practically certain that they would pass over together with the arsenic into the sugar manufactured by means of the impure sulphuric acid, and thus be present in the corresponding beer. Selenium compounds are highly poisonous, and in their physiological action are practically identical with arsenic. Hence these authors conclude that selenium compounds have played a definite rôle in the recent beer-poisoning epidemic, although their part may have been subsidiary to that of arsenic. The moral, of course, is that, in view of the poisonous action of selenium, and the frequency of its presence in those very cases in which arsenic is found, all sulphuric acid used in the manufacture of foods should not only be free from arsenic but free also from selenium.

